# Study Design and Rationale of a Randomized Trial Comparing Aspirin–Sarpogrelate Combination Therapy with Aspirin Monotherapy: Effects on Blood Viscosity and Microcirculation in Cardiovascular Patients

**DOI:** 10.3390/diagnostics15111373

**Published:** 2025-05-29

**Authors:** Yuran Ahn, Jaehyuk Jang, Seonghyeon Bu, Nay Aung, Hyo-Suk Ahn, Keun-Sang Yum

**Affiliations:** 1 Division of Cardiology, Department of Internal Medicine, Uijeongbu St. Mary’s Hospital, College of Medicine, The Catholic University of Korea, 271 Cheonboro, Uijeongbu 11765, Republic of Korea; niceayr@naver.com (Y.A.); let87@naver.com (J.J.); buseonghyeon@gmail.com (S.B.); 2 Cardiovascular Research Institute for Intractable Disease, College of Medicine, The Catholic University of Korea, 222 Banpodaero, Seochogu, Seoul 06591, Republic of Korea; 3 William Harvey Research Institute, Barts and The London School of Medicine and Dentistry, Queen Mary University of London, London E1 2AD, UK; n.aung@qmul.ac.kr; 4 National Institute for Health and Care Research Barts Cardiovascular Biomedical Research Centre, Queen Mary University of London, London E1 4NS, UK; 5 Barts Heart Centre, St Bartholomew’s Hospital, Barts Health NHS Trust, West Smithfield, London EC1A 7BE, UK; 6 Department of Family Medicine, Uijeongbu St. Mary’s Hospital, College of Medicine, The Catholic University of Korea, Seoul 06591, Republic of Korea

**Keywords:** blood viscosity, antiplatelet therapy, sarpogrelate hydrochloride, aspirin, coronary artery disease, peripheral artery disease, flow-mediated dilation, erythrocyte deformability, hemorheology, microcirculation

## Abstract

Coronary artery disease (CAD) and peripheral artery disease (PAD) are associated with increased blood viscosity, which contributes to vascular inflammation and impaired microcirculation. Blood viscosity plays a crucial role in disease progression, influencing endothelial function and tissue perfusion. Sarpogrelate hydrochloride, a serotonin receptor antagonist, has antiplatelet and vasodilatory properties that may improve microvascular function and blood rheology. This randomized, parallel-group, open-label, single-center, phase IV clinical trial enrolled 68 patients with both CAD and PAD. The participants were randomized in a 1:1 ratio to receive either aspirin monotherapy (100 mg) or aspirin (100 mg) plus sarpogrelate (300 mg) for 12 weeks. The primary outcome was the change in blood viscosity from baseline to week 12, assessed using the scanning capillary technique. Secondary outcomes included erythrocyte deformability, flow-mediated dilation (FMD), and tissue oxygen delivery index (tODI), which collectively provide insights into microvascular function and oxygen transport efficiency. Elevated blood viscosity is a key factor in cardiovascular disease progression, yet conventional antiplatelet therapy has shown limited effects on hemorheology. Sarpogrelate, by targeting serotonin-mediated pathways, may enhance microcirculatory function and optimize vascular health. These effects could lead to better oxygen delivery and overall vascular health, thereby optimizing cardiovascular outcomes. By integrating hemorheological and vascular markers, this study aims to provide evidence on the potential benefits of combination therapy. Findings could inform optimized antiplatelet strategies to improve vascular health and reduce cardiovascular risk in patients with CAD and PAD.

## 1. Introduction

Coronary artery disease (CAD) is a condition in which arterial stenosis due to arteriosclerosis leads to impaired myocardial blood flow. It is one of the most significant causes of mortality worldwide and has been increasing in prevalence [1]. Arteriosclerosis is primarily driven by vascular endothelial dysfunction, inflammation within the arterial walls, and plaque formation, processes closely linked to hemodynamic factors [2]. Among these factors, increased blood viscosity has been shown to promote inflammation in the vascular wall, thereby accelerating arteriosclerosis [3]. Several studies have demonstrated a significant association between elevated blood viscosity and the severity of CAD [4,5]. Additionally, changes in erythrocyte deformability and aggregation significantly influence blood viscosity, influencing cardiovascular disease progression and microcirculation [6]. Multiple studies have supported the notion that these hematological factors are closely linked to cardiovascular health and vascular function [7,8].

Antiplatelet agents are essential therapeutic drugs used to inhibit thrombosis in patients and are widely recognized as the standard treatment worldwide [9,10]. Among them, aspirin is the most widely used agent, but its effect on blood viscosity remains unclear. In 2004, Robert S. Rosenson and colleagues evaluated the effects of three different doses of aspirin on whole blood viscosity (WBV) in 100 healthy individuals, finding that aspirin alone did not significantly alter WBV at any dose [11]. In contrast, a 2008 study involving 47 patients with stable cardiovascular disease or high CAD risk compared aspirin monotherapy to a combination of aspirin and dipyridamole, demonstrating that the combination therapy significantly reduced blood viscosity at shear rates of 1 s^−1^ and 2 s^−1^ compared to aspirin alone [12]. These findings highlight the potential advantage of combination therapy in improving blood rheology, thereby supporting the need for further exploration of alternative treatment regimens in cardiovascular disease.

Sarpogrelate hydrochloride—a serotonin receptor antagonist with antithrombotic and vasodilatory properties—is a promising candidate. By targeting serotonin-mediated pathways that drive platelet aggregation and smooth muscle proliferation, it provides a complementary approach to conventional antiplatelet therapies [13]. Evidence suggests that sarpogrelate improves coronary blood flow velocity and microcirculatory function [14,15]. Furthermore, a 2011 study demonstrated that adding sarpogrelate to aspirin therapy reduced platelet aggregation and plasminogen activator inhibitor activity [16]. There was a study to assess the efficacy and safety of dual antiplatlet therapy (DAPT) with sarpogrelate and aspirin compared to clopidogrel and aspirin in PAD patients. Sarpogrelate plus aspirin was found to be non-inferior to clopidogrel plus aspirin in preventing early restenosis after peripheral endovascular therapy [17]. Based on this background, this clinical trial was designed to investigate the effects of sarpogrelate hydrochloride (300 mg) in combination with low-dose aspirin (100 mg). This study aimed to compare aspirin monotherapy and aspirin + sarpogrelate combination therapy in patients with both CAD and PAD, evaluating the potential benefits of sarpogrelate hydrochloride in reducing blood viscosity and improving CAD outcomes. The participants were confirmed to have coronary stenosis ranging from 10% to 75% by coronary angiography or coronary CTA, which are not yet considered indications for interventional procedures.

## 2. Methods

### 2.1. Study Design

**Figure 1:**
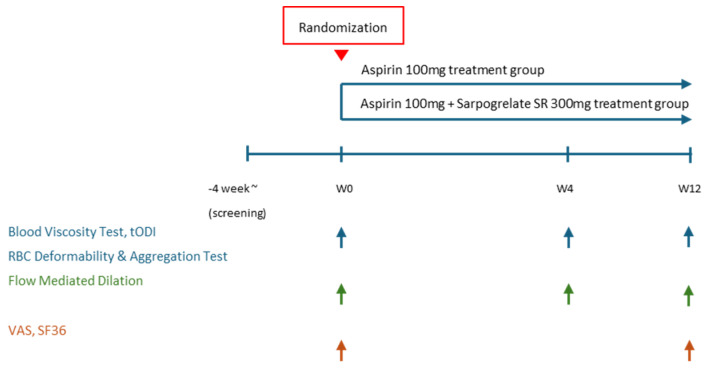
Study flow chart. tODI: tissue oxygen delivery index, VAS: visual analogue scale, SF36: short form 36.

This study was designed as a prospective, randomized, parallel-group, open-label, single-center, phase IV clinical trial. The trial was conducted at Uijeongbu St. Mary’s Hospital, The Catholic University of Korea. The overall study duration included a 12-month recruitment phase including 4 weeks of screening phase and a 3-month treatment period. Assessments were conducted at weeks 4 and 12. A brief flowchart of the whole study is summarized in Figure 1. A detailed breakdown of the study procedures at each visit is provided in Table A1. The investigational products used in this study were Anplag SR Tablets (sustained-release sarpogrelate hydrochloride, 300 mg, Yuhan Corporation, Seoul, Republic of Korea) and Aspirin Protect Tablets (film-coated aspirin, 100 mg, Bayer, Seoul, Republic of Korea). Detailed information on the study treatments is provided in Table A2. Concomitant administration of medications and treatments was permitted if deemed medically necessary based on the judgment of investigators. To minimize bias and improve the reliability of the study findings, standardized protocols were made, and all involved sonographers were trained. Also, we randomly assigned the participants and sonographers when measuring FMD.

### 2.2. Recruitment and Screening

We enrolled patients diagnosed with both peripheral arterial disease (PAD) and coronary artery disease who met the inclusion criteria. Eligible participants were adults aged 19 years or older with coronary artery stenosis of 10 to 75 percent, confirmed by coronary angiography or coronary computed tomography angiography. Patients were also required to have a diagnosis of PAD or exhibit symptoms indicative of the disease. The diagnosis criteria for peripheral arterial disease included those with Bueger’s disease, occlusive arteriosclerosis, diabetic peripheral vascular disease, or those with ischemic symptoms such as peripheral intermittent claudication, ulcers, pain, or cold sensation such as Raynaud’s syndrome. Patients were excluded if they had a planned coronary or cerebrovascular revascularization, recent use of antiplatelet or anticoagulant agents other than aspirin, or severe renal impairment. Those who were previously taking aspirin had a washout period of 2 weeks prior to randomization. Additionally, those with recent major cardiovascular or cerebrovascular events, active bleeding disorders, or pregnancy were not eligible. A detailed summary of the inclusion and exclusion criteria is provided in Appendix A.

### 2.3. Sample Size

**Figure 2:**
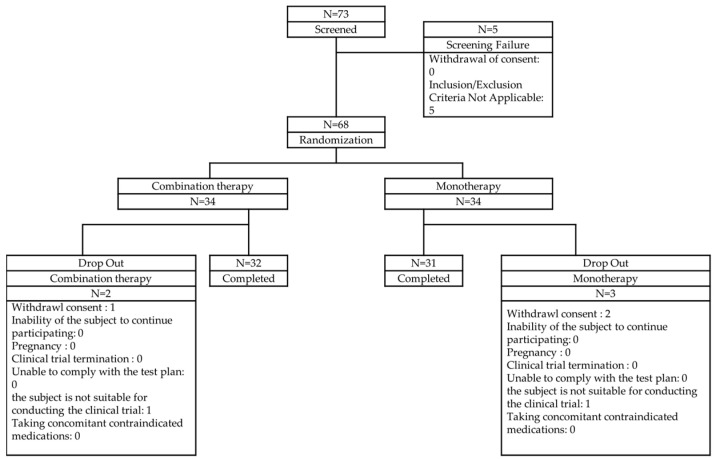
Patient disposition.

As it was difficult to find prior studies directly comparing blood viscosity changes in aspirin and sarpogrelate hydrochloride treatment groups, a previous study comparing blood viscosity between aspirin monotherapy and aspirin + dipyridamole combination therapy was used as a reference. In that study, the change in systolic blood viscosity was reported as 0.10 ± 0.43 cP in the aspirin monotherapy group and 0.22 cP in the combination therapy group. Since the reported difference was not statistically significant, a conservative estimate of 0.43 cP was used for sample size calculation. The significance level was set at α = 0.05 with a statistical power of 80%, and using the designated formula, the required sample size per group was calculated as 27, resulting in a total of 54 participants. Similarly, the change in diastolic blood viscosity was reported as 0.25 ± 1.67 cP in the aspirin monotherapy group and 1.55 cP in the combination therapy group. Again, a conservative approach was taken, applying the same statistical parameters, which resulted in a required sample size of 26 per group, totaling 52 participants. To satisfy both calculations, the final sample size was set at 54 participants. Considering an expected dropout rate of 20%, the final target enrollment was increased to 68 participants (Figure 2).

The participation status of the subjects is shown in Figure 2. Of the 73 subjects who agreed to participate in this clinical trial, 5 were screened out, leaving 68 subjects randomly assigned to 34 in the test group and 34 in the control group. Of the randomly assigned subjects, 2 in the test group (5.88%) and 3 in the control group (8.82%) dropped out, leaving 32 in the test group (94.12%) and 31 in the control group (91.18%) to complete the trial. The most common reason for dropout was the subject’s withdrawal of consent (test group: 2.94%, control group: 5.88%), followed by cases where the subject was deemed unsuitable for participating in the clinical trial (test group: 2.94%, control group: 2.94%).

### 2.4. Demography

The demographic information of 68 safety analysis groups is summarized in Table 1. Among all the participants, 51 individuals (75.00%) were male, and the average age was 6.09 ± 8.23 years. The most common age group was individuals in their 60s, accounting for 31 participants (45.59%). There were no participants who were pregnant, breastfeeding, or of childbearing potential. A total of 38 participants (55.88%) reported alcohol consumption, with an average intake of 91.56 ± 77.67 g per week. In addition, the average height was 163.63 ± 7.77 cm, the average weight was 69.75 ± 10.96 kg, and the BMI was 25.98 ± 3.24 kg/m^2^. In the aforementioned items, there was no statistically significant difference between the test group and the control group (*p* > 0.05). However, in the subjects who smoked, there were 4 people (11.76%) in the test group and 10 people (20.41%) in the control group, and there was a statistically significant difference between the test group and the control group (*p* = 0.0244). The average smoking amount was 16.92 ± 12.91 in the test group and 25.76 ± 20.88 in the control group (*p* = 0.0498).

### 2.5. Randomization and Interventions

Eligible participants were randomly assigned in a 1:1 ratio to either the aspirin monotherapy group or the combination therapy group. Randomization was performed using a computer-generated random sequence, and a central randomization system was used to ensure allocation concealment.

Participants in the monotherapy group received aspirin at a dose of 100 mg once daily for 12 weeks, while those in the combination therapy group received aspirin 100 mg plus sarpogrelate hydrochloride 300 mg once daily for the same duration. Patients on prior aspirin therapy underwent a two-week washout before randomization.

During the treatment period, adherence to medication was monitored through pill counts and self-reported compliance at follow-up visits. The participants were instructed and encouraged to maintain a medication adherence rate of at least 80% throughout the study period. Medication adherence was defined as follows:

Medication adherence (%) = (Number of doses taken)/(Number of doses prescribed) × 100

Safety outcomes were assessed by monitoring the incidence of adverse events and the rate of study drug discontinuation due to adverse events. Additionally, physical examinations, vital sign measurements, and laboratory tests—hemoglobin, platelet, creatinine—were conducted throughout the study period to evaluate anemia.

### 2.6. Outcome Measures

#### 2.6.1. Primary and Secondary Outcomes

The primary outcome measure of this study was the change in blood viscosity from baseline to week 12. This outcome was assessed to evaluate the treatment’s impact on blood rheology.

Secondary outcome measures included the following assessments:

- Change in blood viscosity at week 4 compared to baseline.
- Change in erythrocyte deformability and erythrocyte aggregation at weeks 4 and 12 compared to baseline.
- Change in flow-mediated dilation (FMD) at weeks 4 and 12 compared to baseline.
- Change in oxygen delivery index (tODI) at weeks 4 and 12 compared to baseline.
- Proportion of patients with a ≥20% improvement in tODI at weeks 4 and 12.
- Change in lipid profile, fasting plasma glucose (FPG), homeostatic model assessment for insulin resistance (HOMA-IR), and high-sensitivity C-reactive protein (hs-CRP) at weeks 4 and 12 compared to baseline.
- Change in patient-reported quality of life outcomes (SF-36 and VAS) at week 12 compared to baseline.

#### 2.6.2. Blood Viscosity and Tissue Oxygen Delivery Index

Blood viscosity was measured using the scanning capillary technique with a blood viscometer. This technique scans across the entire range of blood flow velocities and vessel diameters to determine the patient’s blood viscosity.

The tissue oxygen delivery index is defined as the ratio of hematocrit (%) to diastolic blood viscosity (DBV) measured at low shear rates, expressed as follows:

tODI = (Hematocrit (%))/(DBV (cP))

In this clinical trial, DBV for tODI was measured at a shear rate of 5 s^−1^ and a temperature of 37.5 °C. The tODI reflects the efficiency of oxygen delivery by red blood cells and serves as an indicator of tissue perfusion.

#### 2.6.3. RBC Deformability and Aggregation Test

Red blood cell (RBC) deformability is the ability of erythrocytes to change shape to pass through microvessels smaller than their resting diameter. RBC aggregation is a physical parameter that quantifies the tendency of red blood cells to clump together. Elevated aggregation, or a faster aggregation rate, can increase blood viscosity, thereby impairing circulation and contributing directly to the development of cardiovascular and microvascular diseases.

#### 2.6.4. Flow-Mediated Dilation

**Figure 3:**
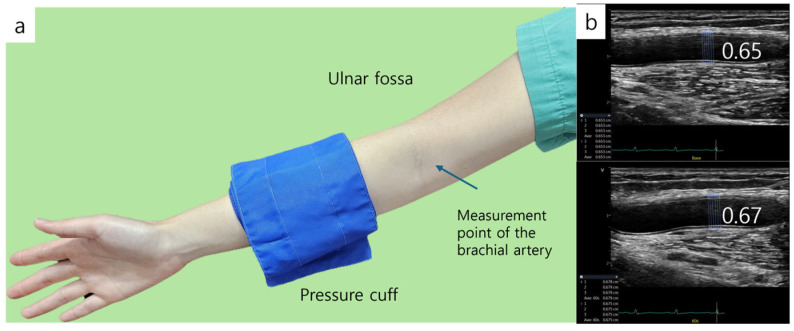
Flow-mediated dilation (FMD). (**a**) Brachial artery diameter is measured at baseline and during hyperemia after inducing 5 min forearm ischemia. Distal location of the cuff. (**b**) FMD is measured, and the diameter of baseline is 0.65 cm and 0.67 cm during hyperemia.

The vascular reactivity of the brachial artery was assessed using the method described by Celermajer and Deanfield [18]. Prior to the examination, subjects rested in a supine position for 10 min to ensure hemodynamic stabilization. After the rest period, high-resolution ultrasound imaging of the right brachial artery was acquired using a 10.0 MHz linear-array transducer. A baseline image of the brachial artery was obtained from the anterior aspect of the antecubital fossa (Figure 3). The image was captured along the long axis, ensuring that the intima layer was clearly visualized at the center. Once an optimal imaging position was determined, the subject’s arm was immobilized and the location was marked. Following baseline imaging, arterial blood flow velocity was measured using pulsed Doppler at a 70° angle relative to the artery. Blood flow was calculated from the cross-sectional area of the vessel and the measured velocity. Endothelium-dependent vasodilation was quantified by the percentage increase in brachial artery diameter during reactive hyperemia. Reactive hyperemia was induced by inflating a cuff on the upper arm to 250 mmHg for 5 min to occlude blood flow. After cuff release, arterial blood flow velocity was recorded for 15 s, and ultrasound imaging continued for up to 90 s. An image corresponding to the R-wave (end-diastole) on the electrocardiogram was selected, stored on the echocardiography system’s hard disk, and later digitized for analysis. The percentage change in arterial diameter relative to the baseline measurement was used to calculate flow-mediated dilation.

The formula for FMD is as follows:

$$\;\frac{\left(maximum\;diameter-baseline\;diameter\right)}{baselinediameter}\times 100\;\left(\%\right)$$

### 2.7. Safety Assessments

The safety endpoints of this study included the incidence of adverse events such as minor bruising to serious hemorrhages—intracranial bleeding, upper and lower GI bleeding, and allergic reaction. The discontinuation rate in this investigation was due to adverse events based on assessments of physical examinations, vital signs, and clinical laboratory tests.

All symptoms and signs during the 12-week (±1 week) treatment period were documented as adverse events. Any pre-existing conditions recorded before administration of the investigational product were classified as medical history rather than adverse events. Adverse events occurring at the final visit or early discontinuation visit were followed up until resolution or until further follow-up was deemed unnecessary. Serious adverse events were defined as events leading to death, life-threatening conditions, hospitalization, persistent disability, or other medically significant complications.

Discontinuation due to adverse events was defined as permanent cessation of the investigational product before the final visit due to adverse reactions. The proportion of participants who discontinued due to adverse events was calculated and analyzed separately for the aspirin monotherapy and combination therapy groups.

### 2.8. Statistical Analyses

#### 2.8.1. Primary Efficacy Endpoint

In order to examine the changes in systolic and diastolic blood viscosity at 12 weeks compared to baseline, descriptive statistics (mean, standard deviation, median, minimum, maximum) for systolic and diastolic blood viscosity at each evaluation time point and administration group and descriptive statistics (mean, standard deviation, median, minimum, maximum) for the changes at 12 weeks compared to baseline were presented. Independent *t*-test or Wilcoxon rank sum test was performed to compare the differences in blood viscosity and changes between administration groups. In addition, a paired *t*-test or Wilcoxon signed-rank test was performed to compare the differences in systolic and diastolic blood viscosity changes within administration groups at 12 weeks compared to baseline. The normality of the data distribution was assessed using the Shapiro–Wilk test. In this clinical trial, the results of blood viscosity tests performed at the screening visit were used as the baseline results.

#### 2.8.2. Secondary Efficacy Endpoint

To evaluate the changes in erythrocyte deformability and aggregation, FMD, tODI, and change rate, lipid profile, FPG, HOMA-IR, hs-CRP, SF-36, and VAS at 4 and 12 weeks will be compared to the baseline, an independent *t*-test or Wilcoxon rank sum test will be performed, and a paired *t*-test or Wilcoxon signed rank test will also be performed to compare differences. Also, the number and proportion of subjects whose tODI improved by 20% or more compared to baseline at 4 and 12 weeks in the administration group will be presented, and a Chi-sqare test or Fisher’s exact test will performed to compare the improvement proportion.

All statistical analyses will be conducted using SAS version 9.4 (SAS Institute, Cary, NC, USA). A significance level of 0.05 will be used for all hypothesis testing, and all *p*-values will be two-tailed.

The efficacy analysis will be conducted using the Full Analysis Set (FAS) and Per Protocol Set (PPS). The FAS will include all randomized participants who receive at least one dose of the study drug and have available data for the primary efficacy endpoint. The PPS will consist of participants who complete the study without major protocol deviations. Safety analyses will be performed on the Safety Set, which will include all participants who receive at least one dose of the study drug and have at least one post-baseline safety assessment.

Baseline characteristics and study outcomes will be summarized using descriptive statistics. Continuous variables will be expressed as means with standard deviations or medians with interquartile ranges, while categorical variables will be presented as frequencies and percentages.

Between-group comparisons of primary and secondary endpoints will use independent *t*-tests or Wilcoxon rank sum tests, depending on data distribution. Within-group changes from baseline will be analyzed using Paired *t*-tests or Wilcoxon signed-rank tests. The proportion of participants achieving predefined thresholds of improvement will be compared between groups using Chi-square tests or Fisher’s exact tests.

For safety analyses, the incidence of adverse events, serious adverse events, and study drug discontinuation due to adverse events will be summarized using descriptive statistics. Between-group comparisons for categorical safety outcomes will be conducted using Chi-square tests or Fisher’s exact tests. Changes in laboratory values, physical examination findings, and vital signs will be evaluated using appropriate statistical tests based on data characteristics.

### 2.9. Regulatory and Ethical Approval

This clinical trial was conducted at Uijeongbu St. Mary’s Hospital, The Catholic University of Korea, with Yuhan Corporation as the trial sponsor. This study was performed in compliance with Good Clinical Practice (GCP), the Declaration of Helsinki, and applicable local regulations, with trial registration number: NCT05730621, date of registration: 26 January 2023. The study protocol, informed consent forms, and all patient-related documentation were reviewed and approved by the Institutional Review Board (IRB) before the initiation of the trial. The trial adhered to all ethical guidelines, ensuring patient safety and confidentiality.

## 3. Discussion

### 3.1. Rationale of the Study and Outcome Measures

This study was designed with blood viscosity as the primary outcome and various vascular and metabolic markers as secondary outcomes, including erythrocyte deformability, erythrocyte aggregation, flow-mediated dilation, and tissue oxygen delivery index.

Blood viscosity has been linked to multiple cardiovascular and cerebrovascular diseases. Elevated whole blood viscosity has been associated with an increased risk of early atherosclerosis in the carotid arteries, incident stroke, and ischemic heart disease [4,19,20]. The Edinburgh Artery Study found that a 0.15 mPas increase in WBV was significantly associated with a higher five-year cardiovascular event risk [20]. Among stroke survivors, those with recurrent cardiovascular events showed significantly elevated WBV at both low and high shear rates [19].

Additionally, one of the secondary outcomes, flow-mediated dilation, has been widely recognized as a non-invasive tool for evaluating endothelial function and assessing cardiovascular risk [21]. The endothelium is the basis of the cardiovascular system and plays a crucial role in vascular homeostasis. To date, prolific data and research have shown evidence that endothelial dysfunction represents the pathophysiological basis for microvascular coronary artery disease and can advance the CAD, and that endothelial dysfunction is a marker that represents CAD prognosis. A decrease in FMD is associated with an increased likelihood of cardiovascular events, making it an important parameter for monitoring vascular health [22]. Erythrocyte deformability and aggregation also play a crucial role in capillary perfusion and oxygen delivery. Impaired erythrocyte deformability has been associated with hypoxia and ischemic damage, contributing to microvascular dysfunction [23,24]. Increased erythrocyte aggregation can further elevate blood viscosity, exacerbating circulatory disturbances in patients with cardiovascular diseases [25]. The Tissue Oxygen Delivery Index reflects microvascular oxygen transport efficiency by integrating hematocrit levels with diastolic blood viscosity. Reduced tODI is linked to impaired tissue perfusion, particularly in conditions such as chronic ischemic disease and diabetes-associated microvascular complications [26].

By incorporating these markers, this study aims to comprehensively evaluate the effects of aspirin–sarpogrelate combination therapy on blood viscosity and related hemorheological parameters. Understanding these effects could help to refine antiplatelet strategies and optimize vascular health management in patients with coronary and peripheral artery disease. Sarpogrelate is a selective serotonin (5-HT_2_A) receptor antagonist that inhibits serotonin-induced platelet aggregation and smooth muscle cell proliferation [27,28]. Serotonin plays a significant role in vascular tone regulation, platelet aggregation, and microvascular circulation [29,30,31]. Serotonin has been shown to stimulate vascular smooth muscle cell proliferation in previous studies [32,33,34]. By blocking serotonin-mediated effects, sarpogrelate has been shown to improve endothelial function and enhance blood flow in both macrovascular and microvascular systems [13,15,35]. Clinical trials have demonstrated its benefits in patients with peripheral artery disease, diabetic microangiopathy, and coronary microvascular dysfunction [14,36,37]. However, its effects on blood viscosity modulation when combined with aspirin remain underexplored. This study aims to fill this gap by evaluating whether sarpogrelate, when added to aspirin therapy, provides additional benefits in improving hemorheological parameters and vascular function.

### 3.2. Limitations and Further Considerations

This study is the first randomized controlled trial designed to assess the effect of aspirin plus sarpogrelate hydrochloride on blood viscosity and vascular function in patients with both PAD and CAD. However, since this is a single-center trial, the generalizability of the findings may be limited. Furthermore, as an open-label study, patient expectations and investigator awareness of treatment allocation could introduce bias, although objective outcome measures help to mitigate this concern. The study duration of 12 weeks may not be sufficient to evaluate long-term cardiovascular outcomes, necessitating further follow-up studies.

Future multi-center trials with extended follow-up are needed to assess the long-term effects of sarpogrelate hydrochloride on clinical outcomes, including major adverse cardiovascular events. Subgroup analyses could help identify patients who may benefit most from this combination therapy, particularly those with elevated baseline blood viscosity or endothelial dysfunction. Additionally, dose optimization studies comparing different doses of sarpogrelate hydrochloride in combination with aspirin may provide further insights into the most effective regimen for improving vascular health.

## Figures and Tables

**Table 1:** Demographic characteristics.

		Combination Group	Monotherapy Group	Total	*p*-Value
		*N* = 34	*N* = 34	*N* = 68	
Gender	Total, *n* (%)	34 (100.00)	34 (100.00)	68 (100.00)	0.7794 †
	Male, *n* (%)	25 (73.53)	26 (76.47)	51 (75.00)	
	Female, *n* (%)	9 (26.47)	8 (23.53)	17 (25.00)	
Age	*n* (%)	34 (100.00)	34 (100.00)	68 (100.00)	0.5796 *
	Mean ± SD	65.53 ± 6.45	66.65 ± 9.75	66.09 ± 8.23	
	Median	66.00	68.00	66.00	
	Min, Max	51.00, 76.00	34.00, 85.00	34.00, 85.00	
Age ranges separated by 10 years	10s, *n* (%)	0 (0.00)	0 (0.00)	0 (0.00)	0.1059 ††
	20s, *n* (%)	0 (0.00)	0 (0.00)	0 (0.00)	
	30s, *n* (%)	0 (0.00)	1 (2.94)	1 (1.47)	
	40s, *n* (%)	0 (0.00)	0 (0.00)	0 (0.00)	
	50s, *n* (%)	6 (17.65)	7 (20.59)	13 (19.12)	
	60s, *n* (%)	20 (58.82)	11 (32.35)	31 (45.59)	
	Above 70s, *n* (%)	8 (23.53)	15 (44.12)	23 (33.82)	
Pregnancy/breastfeeding status	Total, *n* (%)	9 (26.47)	8 (23.53)	17 (25.00)	-
	Yes, *n* (%)	0 (0.00)	0 (0.00)	0 (0.00)	
	No, *n* (%)	9 (26.47)	8 (23.53)	17 (25.00)	
Fertility	Total, *n* (%)	9 (26.47)	8 (23.53)	17 (25.00)	-
	Yes, *n* (%)	0 (0.00)	0 (0.00)	0 (0.00)	
	No, *n* (%)	9 (26.47)	8 (23.53)	17 (25.00)	
Smoking	Total, *n* (%)	34 (100.00)	34 (100.00)	68 (100.00)	0.0244 †
	Never, *n* (%)	8 (23.53)	13 (38.24)	21 (30.88)	
	Current smoker, *n* (%)	4 (11.76)	10 (29.41)	14 (20.59)	
	Ex-smoker, *n* (%)	22 (64.71)	11 (32.35)	33 (48.53)	
Amount of smoking (cigarette/day)	*n* (%)	25 (73.53)	21 (61.76)	46 (67.65)	0.0498 **
	Mean ± SD	16.92 ± 12.91	25.76 ± 20.88	20.96 ± 17.39	
	Median	13.00	20.00	20.00	
	Min, Max	2.00, 60.00	3.00, 100.00	2.00, 100.00	
Alcohol	Total, *n* (%)	34 (100.00)	34 (100.00)	68 (100.00)	0.8584 †
	Never, *n* (%)	11 (32.35)	9 (26.47)	20 (29.41)	
	Current, *n* (%)	18 (52.94)	20 (58.82)	38 (55.88)	
	Past, *n* (%)	5 (14.71)	5 (14.71)	10 (14.71)	
Alcohol (g/week)	*n* (%)	23 (67.65)	25 (73.53)	48 (70.59)	0.7178 **
	Mean ± SD	84.35 ± 45.31	98.20 ± 99.18	91.56 ± 77.67	
	Median	70.00	70.00	70.00	
	Min, Max	20.00, 140.00	10.00, 420.00	10.00, 420.00	
Height (cm)	*n* (%)	34 (100.00)	34 (100.00)	68 (100.00)	0.4112 *
	Mean ± SD	164.41 ± 6.85	162.85 ± 8.62	163.63 ± 7.77	
	Median	165.50	162.00	164.25	
	Min, Max	150.00, 177.00	138.00, 180.00	138.00, 180.00	
Weight (kg)	*n* (%)	34 (100.00)	34 (100.00)	68 (100.00)	0.5487 *
	Mean ± SD	70.56 ± 8.16	68.95 ± 13.26	69.75 ± 10.96	
	Median	72.00	69.90	70.00	
	Min, Max	55.00, 84.00	48.00, 98.50	48.00, 98.50	
BMI (kg/m^2^)	*n* (%)	34 (100.00)	34 (100.00)	68 (100.00)	0.7415 *
	Mean ± SD	26.11 ± 2.82	25.85 ± 3.64	25.98 ± 3.24	
	Median	25.65	26.10	25.95	
	Min, Max	21.10, 33.30	19.10, 34.50	19.10, 34.50	

* Independent *t*-test; ** Wilcoxon rank sum test; † Chi-Square test; †† Fisher’s exact test.

## Data Availability

The clinical study data are confidential and belong to Yuhan Corporation. The dataset supporting the findings of this study is not publicly available but may be accessed upon reasonable request and approval from the sponsor.

## Appendix A

**Table A1:** Study flow chart.

Procedure	Screening (<4 Weeks)	Randomization (Week 0)	Treatment (Week 4 ± 1 Week)	Treatment (Week 12 ± 1 Week)	Early Discontinuation
Visit	SV	Visit 1	Visit 2	Visit 3	ED
Informed consent	✓				
Eligibility assessment	✓				
Medical history and demographics	✓				
Physical examination	✓	✓	✓	✓	✓
Vital signs	✓	✓	✓	✓	✓
Laboratory tests	✓	✓	✓	✓	✓
Randomization		✓			
Study drug administration		✓	✓	✓	
Concomitant medication assessment	✓	✓	✓	✓	✓
Medication adherence assessment		✓	✓	✓	
Adverse event monitoring		✓	✓	✓	✓
Early discontinuation assessment					✓

**Table A2:** Study treatment details.

Product Name	Manufacturer	Dosage Form and Appearance	Active Ingredient	Storage Conditions
Anplag SR Tablets 300 mg	Yuhan Corporation	White, film-coated, sustained-release tablet	Sarpogrelate hydrochloride 300 mg	Sealed container, store at 1–30 °C
Aspirin Protect Tablets 100 mg	Bayer Korea	White, round, film-coated tablet	Aspirin 100 mg	Sealed container, store at 1–25 °C

### Inclusion and Exclusion Criteria

Inclusion Criteria

- Patients aged 19 years or older at the time of informed consent.
- Individuals diagnosed with coronary artery stenosis of 10–75% confirmed by coronary angiography or coronary computed tomography angiography.
- Individuals diagnosed with peripheral arterial disease (PAD) or exhibiting symptoms of PAD.
- Individuals who have fully understood the study objectives, the characteristics and risks of the investigational drug, and have provided written informed consent after receiving a thorough explanation.

*PAD diagnostic criteria: Individuals diagnosed with thromboangiitis obliterans, atherosclerotic occlusive disease, or diabetic peripheral vascular disease, or those presenting with ischemic symptoms such as intermittent claudication, ulcers, pain, or cold sensation (Raynaud’s phenomenon).

Exclusion Criteria

- Individuals scheduled for surgery related to coronary artery disease or cerebrovascular disease.
- Individuals who have taken aspirin within two weeks prior to randomization.
- Individuals currently receiving or planning to receive antiplatelet agents or anticoagulants other than aspirin.
- Individuals with the following laboratory test results at the time of screening:
  - Hemoglobin level below 13 g/dL (or below 12 g/dL for females).
  - Platelet count (PLT) below 60,000/µL.
  - Severe renal impairment (eGFR < 30 mL/min/1.73 m^2^, based on the CKD-EPI equation).
- Individuals with a history of cerebrovascular or cardiovascular complications (stroke, transient ischemic attack, myocardial infarction, unstable angina, coronary artery bypass grafting, or percutaneous coronary intervention) within the past six months.
- Individuals with active bleeding disorders (including hemophilia, capillary fragility syndrome, gastric ulcer bleeding, urinary tract bleeding, hemoptysis, or vitreous hemorrhage).
- Pregnant or lactating women, or those planning to become pregnant.
- Individuals currently participating in another clinical trial involving investigational drugs.
- Any other individuals deemed inappropriate for participation in this clinical trial by the investigator.
